# *Arabidopsis thaliana* eIF4E1 and eIF(iso)4E Participate in Cold Response and Promote Translation of Some Stress-Related mRNAs

**DOI:** 10.3389/fpls.2021.698585

**Published:** 2021-09-30

**Authors:** Kenia Salazar-Díaz, Mayra Aquino-Luna, Eloísa Hernández-Lucero, Brenda Nieto-Rivera, Marlon A. Pulido-Torres, Jesús H. Jorge-Pérez, Marina Gavilanes-Ruiz, Tzvetanka D. Dinkova

**Affiliations:** Departamento de Bioquímica, Facultad de Química, Universidad Nacional Autónoma de México, Mexico City, Mexico

**Keywords:** Arabidopsis, cold acclimation, eIF4E, eIF(iso)4E, freezing tolerance, polysomes

## Abstract

Plant defense and adaptation to adverse environmental conditions rely on gene expression control, such as mRNA transcription, processing, stability, and translation. Sudden temperature changes are common in the era of global warming; thus, understanding plant acclimation responses at the molecular level becomes imperative. mRNA translation initiation regulation has a pivotal role in achieving the synthesis of the appropriate battery of proteins needed to cope with temperature stress. In this study, we analyzed the role of translation initiation factors belonging to the eIF4E family in Arabidopsis acclimation to cold temperatures and freezing tolerance. Using knockout (KO) and overexpressing mutants of *AteIF4E1* or *AteIF(iso)4E*, we found that AteIF4E1 but not AteIF(iso)4E overexpressing lines displayed enhanced tolerance to freezing without previous acclimation at 4°C. However, KO mutant lines, *eif(iso)4e-1* and *eif4e1-KO*, were more sensitive to the stress. Cold acclimation in wild-type plants was accompanied by increased levels of *eIF4E1* and *eIF(iso)4E* transcript levels, polysomes (P) enrichment, and shifts of these factors from translationally non-active to active fractions. Transcripts, previously found as candidates for eIF(iso)4E or eIF4E1 selective translation, changed their distribution in both P and total RNA in the presence of cold. Some of these transcripts changed their polysomal distribution in the mutant and one eIF4E1 overexpressing line. According to this, we propose a role of eIF4E1 and eIF(iso)4E in cold acclimation and freezing tolerance by regulating the expression of stress-related genes.

## Introduction

As sessile organisms, plants have developed a wide range of strategies to strengthen their endurance to a changing surrounding ambient [reviewed in: Haak et al. (2017)]. Some defense mechanisms rely on gene expression control, encompassing regulation of mRNA transcription, processing, stability, and translation. Tight regulation at several levels allows plants to readjust their entire metabolism, growth rate, and development and to promote their survival capacity (Chinnusamy et al., 2010; Echevarria-Zomeno et al., 2013; Ding et al., 2020).

Cold stress is common to plants growing in a temperate climate. In their habitat, the freezing season follows previous exposure to cold non-freezing temperatures. This process is known as cold acclimation and allows freezing tolerance acquisition (Thomashow, 1999). *Arabidopsis thaliana* has been an important model to study the acquired freezing tolerance through cold acclimation. Events occurring during cold responses include the modification of plasma membrane fluidity, cytoskeleton rearrangement, rise of intracellular calcium concentration, and the activation of kinase cascades that trigger specific gene expression patterns (Guo et al., 2018; Ding et al., 2020). Particularly, a set of cold-regulated transcription factors orchestrate the production of mRNAs corresponding to proteins responsible for membrane stability, osmotic equilibrium maintenance, antioxidant mechanisms activation, and the cold signaling cascade itself (Chinnusamy et al., 2007; Novillo et al., 2007).

Besides transcriptional readjustment, post-transcriptional control of gene expression becomes very important in stress response or acclimation. Intron retention, alternative splicing, and microRNA-mediated mRNA decay are some of the mechanisms with demonstrated roles under these conditions (Guerra et al., 2015). Although fewer reports deal with the control of translation under abiotic stress, there is growing evidence that this is a key event in plant response. Genome-wide analyses have unraveled protein synthesis changes and the upregulated translation of specific stress response factors under various stress conditions in Arabidopsis and other plant species (Branco-Price et al., 2005; Matsuura et al., 2010; Yanguez et al., 2013; Garcia-Molina et al., 2020). While the mechanisms underlying such regulation remain mostly unknown, it has been proposed that RNA-binding proteins and translation components might contribute to selective mRNA translation under stress (Juntawong et al., 2013; Beine-Golovchuk et al., 2018; Lokdarshi et al., 2020). Noticeably, significant upregulation of ribosome biogenesis and translation initiation factors was reported in Arabidopsis transcriptome after 10-day cold exposure, suggesting that protein synthesis is enhanced under prolonged cold (Xi et al., 2020).

Control can be exerted at all steps of the translation process, but the initiation phase is particularly relevant [reviewed in: Bailey-Serres et al. (2009)]. This step involves the recognition of mRNA 5'end (5'cap) by eIF4E bound to the multi-domain protein eIF4G (eIF4F complex) to recruit several other factors and the small 40S subunit (Browning and Bailey-Serres, 2015). In plants, eIF4E and eIF4G form the eIF4F complex, while eIF(iso)4E and eIF(iso)4G form the eIF(iso)4F complex. Both complexes support general translation and have partially redundant functions. However, each one can also contribute to selective translation from a set of mRNAs (Dinkova et al., 2011; Martinez-Silva et al., 2012).

Little is known about how particular mRNAs become preferentially translated under stress conditions. Informatic analyses have pointed at mRNA features, such as the GC content and length of the 5' untranslated region (5'UTR), the presence of upstream open reading frames (uORFs), Internal Ribosome Entry Site (IRES), and RNA-binding motifs (Dinkova et al., 2005; Kawaguchi and Bailey-Serres, 2005; Matsuura et al., 2013; Von Arnim et al., 2014). However, a link between these characteristics and the translation machinery is still missing.

The presence of different family members for eIF4E and eIF4G was early proposed to account for selective mRNA translation in plants (Kawaguchi and Bailey-Serres, 2002). Although their differential role resulted more evident in viral infection of many plant species (Robaglia and Caranta, 2006), particular functions were found during normal plant development (Martinez-Silva et al., 2012). In *Arabidopsis thaliana*, the *eIF4E1* transcript is expressed ubiquitously in all plant organs, whereas *eIF(iso)4E* is more abundant in roots and flowers (Rodriguez et al., 1998). At the protein level, both factors are enriched in proliferative cells supporting their role in general translation (Bush et al., 2009). Knockout (KO) eIF(iso)4E Arabidopsis mutant plants are viable and display normal phenotypes but present substantially increased eIF4E1 protein levels (Duprat et al., 2002). Microarray analysis of mRNA distribution along polysomal profiles allowed the identification of specific transcripts altered in their translational status in this mutant (Martinez-Silva et al., 2012). Among the differentially translated mRNAs, there was an enrichment of stress-regulated transcripts suggesting that the ratio of eIF4E1/eIF(iso)4E might be relevant in stress response.

In this study, we tested the role of these isoforms in cold acclimation and freezing tolerance of Arabidopsis adult plants, using the *ateif(iso)4e-1* KO mutant and AteIF4E1 or AteIF(iso)4E overexpressing lines as compared to the wild type (Col-0). The AteIF4E1 overexpressing lines displayed enhanced freezing tolerance without previous acclimation at 4°C. Furthermore, cold acclimation in Col-0 was accompanied by increased eIF4E1 and eIF(iso)4E transcript levels and changes in polysomal profiles. By testing the polysomal distribution and total RNA levels of transcripts, previously found as candidates for eIF(iso)4E or eIF4E1 preferential recruitment, we propose a role of these proteins in cold acclimation and freezing tolerance by translational regulation of stress-related genes.

## Materials and Methods

### Plant Material

All plants were *Arabidopsis thaliana* Columbia ecotype (Col-0). The eIF(iso)4E mutant line, *eif(iso)4e-1*, was provided by Dr. Christophe Robaglia from CNRS-CEA Université de la Mediterranée, France (Duprat et al., 2002). The eIF4E1 KO line, *eif4e1-KO*, was donated by Dr. Jean Luc Gallois from INRA, Montfavet, France (Bastet et al., 2018). Transgenic lines expressing AteIF(iso)4E or AteIF4E1 under the constitutive Cauliflower Mosaic Virus (CaMV) 35S promoter, *eIF(iso)4E-OE*, and *eIF4E1-OE* were generated by substituting the β-glucuronidase (GUS) sequence in pBI121 vector (Jefferson et al., 1987) for the coding region of *eIF(iso)4E* or *eIF4E1* (only exons) and Agrobacterium-mediated transformation of Col-0 plants. Overexpressing lines were selected by Kanamycin resistance and verified at the DNA level with primers for each construct [*35S::eIF4E1* and *35S::eIF(iso)4E*]. Three to four independent homozygous lines were isolated by self-fertilization for at least four generations prior to use.

### Growth Conditions and Freezing Treatment

Seeds were sown on Metromix-200 substrate and incubated at 22°C in a growth chamber under an 8 h light/16 h darkness regime for 5 or 7 weeks. After this, half of the plants were transferred to acclimation (4 ± 1°C, acclimated, AC) under the same photoperiod for an additional week. The other half was left under 22°C (non-acclimated, NAC). Phenotypes were registered for AC and NAC plants freezing. Stress was applied in a low-temperature chamber at −20°C for 1 h in the dark, followed by a short recovery phase at 4 ± 1°C for 40 min. Finally, the plants were left for long recovery (4 weeks) at 22°C under 8 h light/16 h darkness regime. The phenotypes under recovery and survival percentages were registered at 2 and 4 weeks upon the freezing stress. All experiments were performed for 3–5 independent biological replicates.

### RNA Accumulation Measurements by RT-qPCR

Total RNA was extracted from the middle rosette leaves of AC and NAC plants by using the TRizol reagent. Upon verification of RNA integrity by agarose gel electrophoresis, 5 μg of RNA was treated with DNase RQ1 (Promega, Madison, WI, USA) in a 50 μl reaction and purified with the RNA Clean and Concentrator kit (Zymo Research, Irvine, CA, USA) following the instructions of the manufacturer. Quantitative RT-qPCR was performed with SuperScript III Platinum SYBR Green One-Step kit (Invitrogen) and gene-specific primers (Supplementary Table 1). Relative expression was calculated by the 2^−ΔΔCt^ method (Pfaffl, 2001) considering NAC as the reference sample and actin or rRNA 18S as the normalizing transcript. Three independent RNA analyses were performed for each one of two different biological experiments.

### Protein Extraction and Western Blot Analysis

Total protein was extracted from leaves of AC and NAC plants in extraction buffer consisting of 100 mM hydroxyethyl piperazineethanesulfonic acid (HEPES) having pH value 7.5, 5% v/v glycerol, 50 mM KCl, 5 mM ethylenediaminetetraacetic acid (EDTA), 0.1% v/v Triton X-100, 1 mM dithiothreitol (DTT), and complete EDTA-free protease inhibitor cocktail (Merck, Darmstadt, Germany). The extracts were centrifuged at 13,000 *g* for 15 min to eliminate cell debris, and the protein concentration was determined in clear supernatants with the Bradford reagent (Bio-Rad Laboratories, Inc. Hercules, CA). Twenty-five micrograms of total protein was separated by sodium dodecyl sulfate–polyacrylamide gel electrophoresis (SDS-PAGE), blotted onto polyvinylidene fluoride (PVDF) membrane (Merck, Darmstadt, Germany), and blocked with 5% (w/v) non-fat milk in PBS-Tween. Blots were incubated with antibodies against AteIF4E1 and AteIF(iso)4E (donated by Dr. Karen Browning, the University of Texas at Austin, TX) and secondary antibodies at 1:10,000 dilutions each, for 2 h at room temperature. Signals were detected with Immobilon Western Chemiluminescent HRP Substrate (Merck, Darmstadt, Germany).

### Polysomal Profiling and RNA Analysis

Two grams of middle rosette leaves was crushed with liquid nitrogen and homogenized in 3.5 ml of lysis buffer consisting of 200 mM Tris-HCl (pH 8.5), 50 mM KCl, 25 mM MgCl_2_, 2 mM ethylene glycol tetraacetic acid (EGTA), 2% PTE (10-Tridecyl Polyoxyethylene ether), 1% octylphenoxy poly(ethyleneoxy)ethanol (IGEPAL), 1% Tritón X-100, 0.1% DTT (1M), 0.2 mg/ml cycloheximide, and 0.5 mg/ml Heparin. After the lysis, samples were centrifuged twice at 12,000 g at 4°C for 15 min. The supernatant was placed on 60% sucrose in gradient buffer containing 50 mM Tris-HCl (pH8.5), 20 mM KCl, 10 mM MgCl_2_, and 0.2 mg/ml cycloheximide and centrifuged in a Beckman 80Ti rotor at 50,000 rpm at 4°C for 3 h. The pellet was dissolved in 400 μl of lysis buffer, layered onto 20–60% sucrose continuous gradient, and centrifuged in a Beckman SW55Ti rotor at 45,000 rpm at 4°C for 2 h. The gradient was fractionated with an ISCO collector and continuous monitoring of the absorbance at 260 nm. RNA isolation from each fraction was carried out according to Martinez-Silva et al. (2012). DNase treatment and RNA purification were performed as described for total RNA analysis. For the RNA obtained from each fraction, reverse transcription was performed with Improm-II Reverse Transcriptase (Promega, Madison, WI, USA), and equal volumes of cDNA were amplified with gene-specific primers from each fraction separately (end-point PCR) or pooled fractions corresponding to non-polysomes (NP) and polysomes (P) by RT-qPCR. One profile was analyzed by end-point RT-PCR for all genes. In a second biological replicate, *eIF4E, eIF(iso)4E, TCF1*, and *COR15A* were analyzed for reproducibility in their overall profile distribution. The results from end-point PCR were evaluated by densitometry to represent the RNA corresponding to each fraction as a percentage from the sum of all fractions. Two profiles corresponding to independent biological replicates for Col-0, *eif(iso)4e-1*, and *eIF4E1-OE-1* plants were analyzed by RT-qPCR. Results from RT-qPCR were reported as mean ratios of mRNA abundance in P/NP for each line and condition. Further, the fold change of the P/NP ratio found in AC plants as compared to that of NAC plants was calculated.

### Statistical Analysis

For transcript or protein levels, Student's *t*-test was used to evaluate statistically significant differences with respect to control; *p* < 0.05. For phenotype evaluations and P/NP ratios in different lines and conditions, comparisons were performed using one-way ANOVA followed by the Dunnett test; *p* < 0.05.

## Results

The translation process is tightly regulated during plant abiotic stress response to promote strictly selective protein synthesis. However, selectivity mechanisms are poorly understood. Initiation factors belonging to the eIF4E family have been described as main targets for general and selective translational control in animal responses to environmental signals. In this study, we analyzed the eIF4E1 and eIF(iso)4E function in the protection of *Arabidopsis thaliana* against cold injury.

### Expression of eIF4E1 and eIF(iso)4E Is Stimulated by Low Temperatures

**Figure 1:**
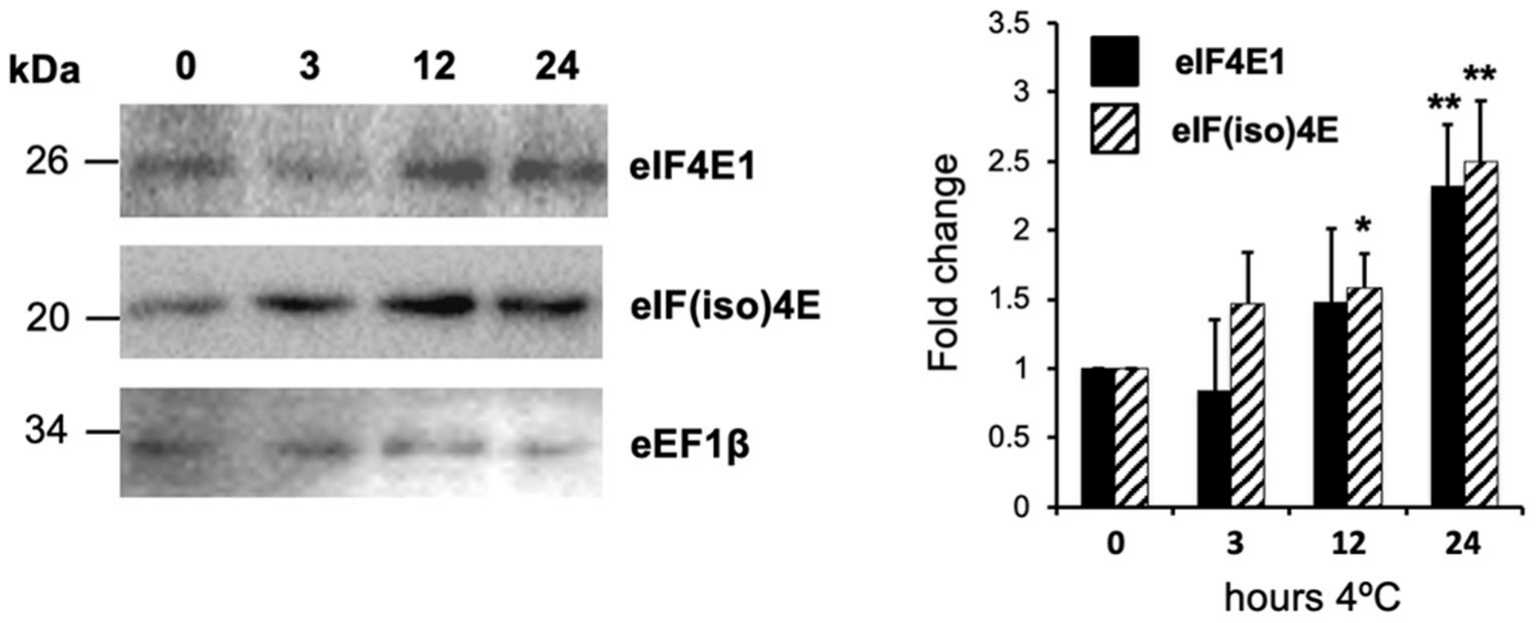
Translation factors eIF4E1 and eIF(iso)4E expression is upregulated by low temperatures. Two weeks old plantlets were placed in a cold chamber at 4°C under a 16 h light/8 h darkness photoperiod (long day) for 24 h. Protein levels were evaluated by Western blot and translation elongation factor eEF1β was used for normalization. The graph at the right represents protein level measurements by blot densitometry of three replicates. Data are presented as mean fold change with respect to control plants before cold exposure. Student's *t*-test was used to evaluate statistically significant differences: ^*^p < 0.05, ^**^p < 0.01.

Global expression data for different abiotic stresses applied to Arabidopsis plants are available at the Arabidopsis eFP Browser—www.bar.utoronto.ca (Winter et al., 2007). According to these, the expression of *eIF4E1* (*At4g18040*) and *eIF(iso)4E* (*At5g35620*) increases 1.6 and 1.3 times, respectively, after 24 h of incubation at 4°C (Supplementary Figure 1). The earlier response is observed for *eIF4E1*, but both transcripts display a cyclic behavior between 0.5 and 24 h of exposure at 4°C. However, it is not known whether the protein levels mirror the mRNA expression patterns under these conditions. Therefore, we assayed a similar cold treatment on 15-day-old plantlets and estimated protein levels for both translation factors in whole plantlet tissues. Both factors showed significantly higher levels as proteins, reaching increases that were higher than two-fold at 24 h of cold exposure (Figure 1). eIF(iso)4E displayed an earlier accumulation response as compared to eIF4E1. Such behavior suggests that both, eIF4E1 and eIF(iso)4E, could function in the cold response of Arabidopsis, although their expression regulation under these conditions might differ. To explore the effect of different levels of eIF4E1 and eIF(iso)4E on the plant response to more aggressive cold conditions, we imposed freezing temperatures on adult plants lacking eIF(iso)4E, i.e., using the KO mutant: *eif(iso)4e-1* (Duprat et al., 2002; Martinez-Silva et al., 2012) or overexpressing either eIF4E1 or eIF(iso)4E under the constitutive 35S CAMV promoter. The mRNA and protein levels of each factor were estimated in the different Arabidopsis transgenic lines (Supplementary Figure 2), and initial phenotypes were registered for several overexpressing lines (Supplementary Figure 3). Lines showing consistently higher levels for eIF4E1 (*eIF4E1-OE-1*) or eIF(iso)4E (*eIF(iso)4E-OE-6*) were selected to test in several independent biological replicate experiments.

### Translation Initiation Factors eIF4E1 and eIF(iso)4E Protect Plants Against Freezing

**Figure 2:**
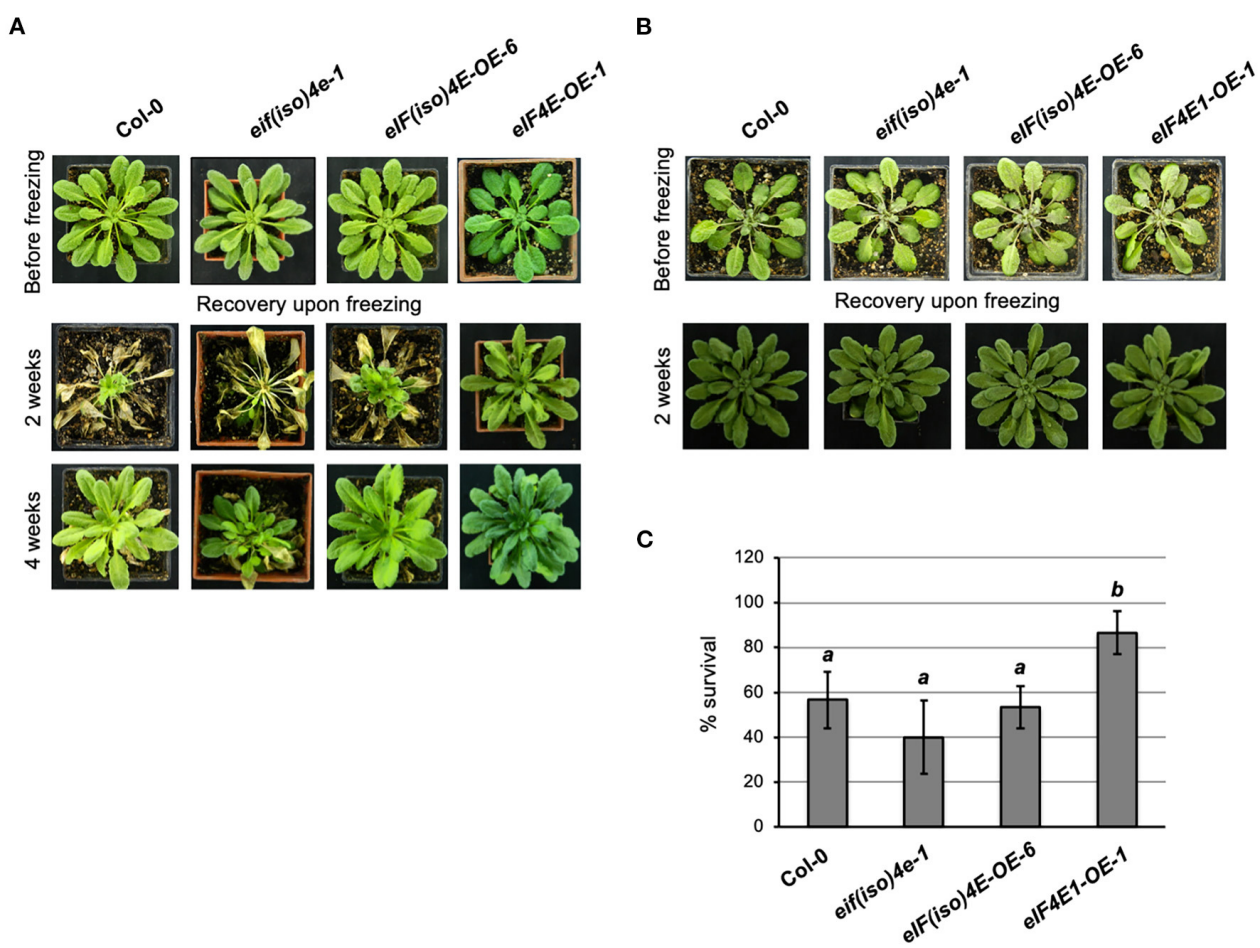
eIF4E1 overexpression enhances *Arabidopsis thaliana* freezing tolerance in adult non-acclimated plants. Plant phenotypes were registered before freezing, and at 2 or 4 weeks after stress recovery. Col-0, wild type; *ateif(iso)4e-1*, knockout *eIF(iso)4E* mutant; *AteIF(iso)4E-OE-6*, plants expressing eIF(iso)4E under the CaMV 35S promoter; *AteIF4E1-OE-1*, plants expressing eIF4E1 under the CaMV 35S promoter. **(A)** Adult 8-week-old plants were grown at 22°C under 8 h light/16 h darkness photoperiod (short day) and placed, without previous acclimation, under freezing temperatures (−20°C) for 1 h. **(B)** Adult 7-week-old plants were grown at 22°C and for an additional week at 4°C (acclimation) under 8 h light/16 h darkness photoperiod (short day) previous to freezing temperature (−20°C) exposure for 1 h. **(C)** Percentage of plant survival upon freezing without acclimation. Analysis was performed using one-way ANOVA followed by the Dunnett test; *p* < 0.05.

Freezing temperature (−20°C) was first applied for 1 h in the dark to 8-week-old plants grown under standard temperature conditions without acclimation at 4°C (NAC). Interestingly, surviving plants overexpressing the eIF4E1 factor displayed a healthier aspect compared to all other lines used in the study (Figure 2 and Supplementary Figure 3). The phenotype was particularly distinctive at 2 weeks after stress. Upon 4 weeks of recovery, the phenotype of surviving plants was similar for Col-0 and the overexpressing lines. However, the *eif(iso)4e-1* mutant line was still injured even after 4 weeks of recovery upon freezing. The recorded phenotype of plants that survived freezing in three independent experiments showed a reproducible behavior. The survival capacity resulted significantly higher only for the eIF4E1 overexpressing line (Figure 2C). Curiously, the *eif(iso)4e-1* mutant line showed a lower but not significantly different plant survival rate as compared to the wild type (Col-0). Since *eif(iso)4e-1* displayed higher eIF4E1 protein levels (Duprat et al., 2002; Martinez-Silva et al., 2012), it could be concluded that such overexpression was not sufficient to improve the basal freezing tolerance of the adult plant in the absence of eIF(iso)4E.

To evaluate the role of eIF4E factors during cold acclimation, freezing was assayed on plants previously AC for 1 week at 4°C. Acclimation was applied to 7-week-old plants for completing 8 weeks at the time of freezing stress. All lines showed a 100% survival rate upon freezing, and no distinguishable phenotypes were recorded after 2 weeks of recovery in all replicate experiments (Figure 2B). This result indicated that plants were able to achieve adequate freezing tolerance upon acclimation, even in the absence of eIF(iso)4E.

**Figure 3:**
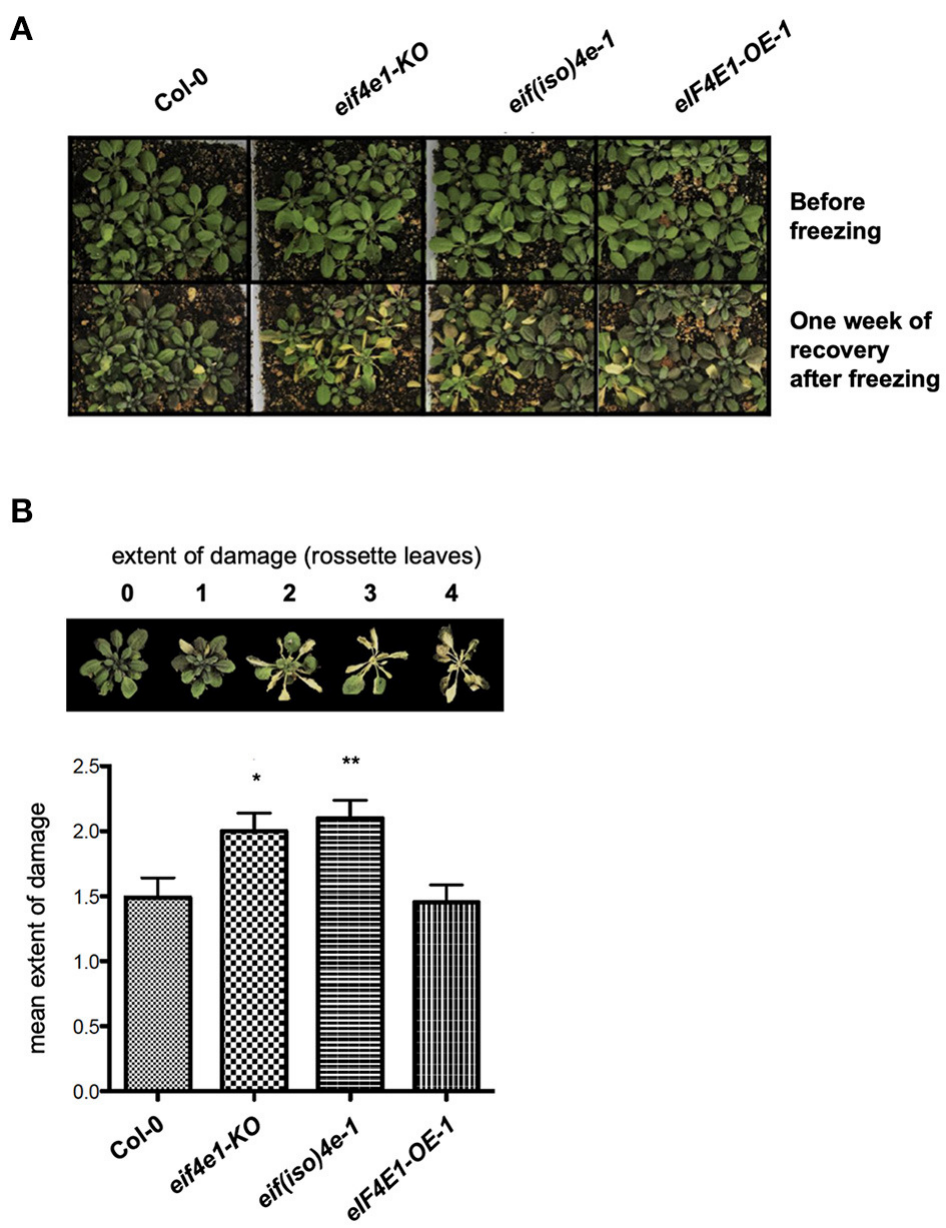
The absence of eIF4E1 or eIF(iso)4E reduces the freezing tolerance of 5-week-old Arabidopsis plants. **(A)** Plant phenotypes were registered before freezing, and after 1 week of recovery upon freezing exposure for 1 hour. Col-0, wild type; *ateif(iso)4e-1*, knockout *eIF(iso)4E* mutant; *eIF4E1-KO*, knockout *eIF4E1* mutant; *AteIF4E1-OE*, plants expressing eIF4E1 under the CaMV 35S promoter. **(B)** Mean values of damage extent were calculated after registering the phenotype of 60 plants per line in five biological replicate experiments, according to the scale shown above the graph. Significant differences were evaluated by one-way ANOVA followed by Dunnett test; ^*^p < 0.05, ^**^p < 0.01.

Next, we tested the effect of eIF(iso)4E or eIF4E1 absence and eIF4E1 overexpression on younger plants (5 weeks old) subjected to 1 h freezing stress under NAC conditions. At this stage, a larger number of plants were simultaneously assayed (20 plants per line, per experiment) in five independent biological replicates (Figure 3A). The extent of damage was established as follows: no damaged leaves (0); 25% damaged leaves (1); 50% damaged leaves (2); 75% damaged leaves (3); and 100% damaged leaves (4). In this way, the average damage extent was calculated after registering the phenotype at 1 week of plant recovery. The absence of eIF4E1 or eIF(iso)4E significantly affected the response of 5-week-old NAC plants to freezing when compared to the Col-0 background (Figure 3B). However, eIF4E1 overexpression did not show significant protection at this stage. While juvenile plants are possibly more tolerant to freezing than adult plants, even in the absence of acclimation (Livingston et al., 2018), the role of eIF4E1 and eIF(iso)4E exerted during cold stress might also differ depending on the developmental stage, according to their endogenous level changes during growth (Bush et al., 2009).

### Transcriptional Adjustments of Cold-Responsive Genes Upon Acclimation

**Figure 4:**
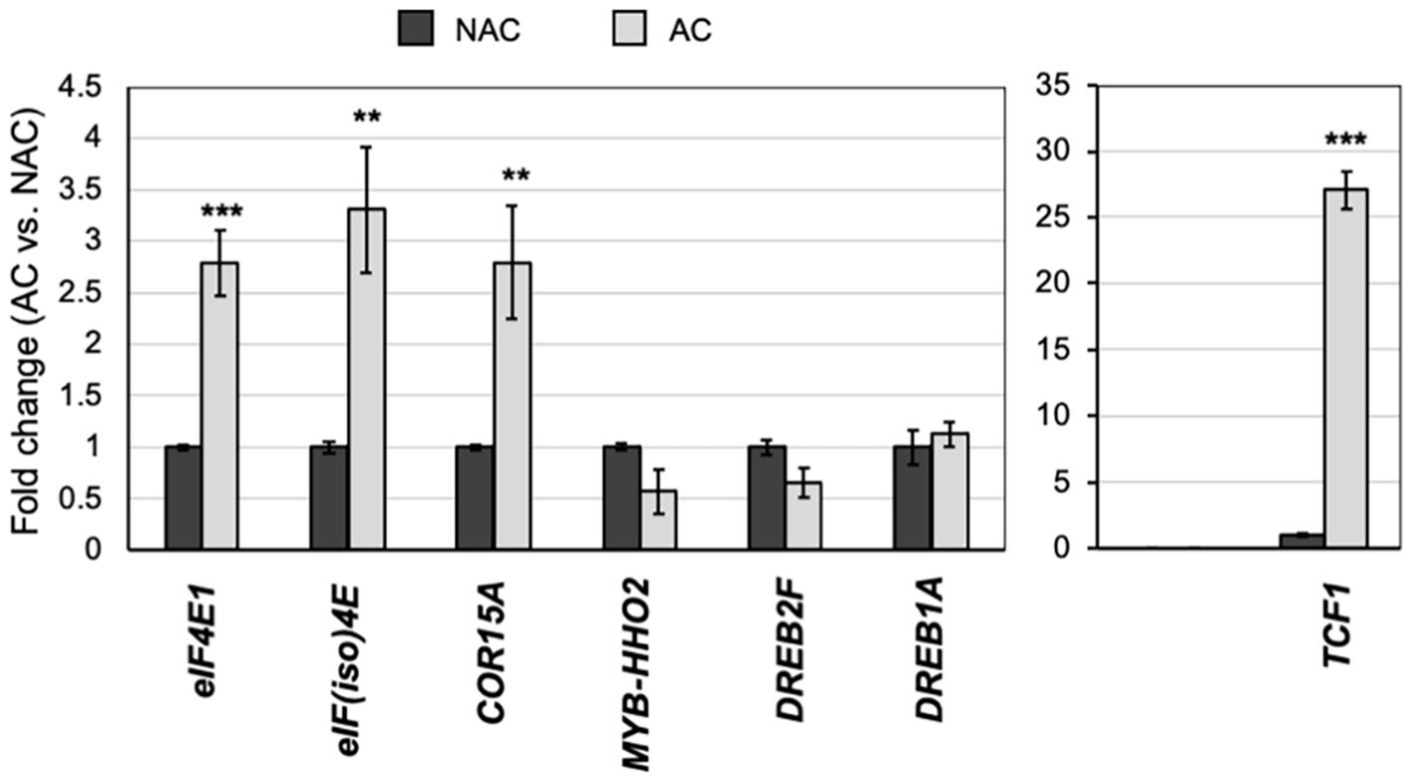
Expression of stress-responsive genes upon cold acclimation in wild-type Col-0 plants. Transcript levels of selected stress-responsive genes (see Supplementary Figure 4), *eIF4E1*, and *eIF(iso)4E* were analyzed by RT-qPCR in acclimated (AC; gray bars) and non-acclimated (NAC; black bars) 8-week-old adult plants grown under short photoperiod. Actin was used as reference control and plotted values represent the expression of each mRNA relative to NAC. Three independent replicates were analyzed. Student's *t*-test was used to evaluate statistically significant differences: ^**^p < 0.01, ^***^p < 0.001.

Arabidopsis plants possess acclimation mechanisms that protect them from very low temperatures. During the acclimation period to mild low temperatures (4°C), upregulation of cold-response genes takes place to physiologically prepare the plant cells for cold resistance. While cap-binding factors eIF4E1 and eIF(iso)4E are relevant for translation, their regulatory role extends to other RNA metabolism features, such as stability and subcellular localization. This could intimately interact with transcriptional regulation by adjusting transcription factors mRNA accumulation and translation. On a previously reported global screen for translationally affected mRNAs in the *eif(iso)4e-1* mutant (Martinez-Silva et al., 2012), we identified a group of cold-regulated genes by using the Arabidopsis eFP Browser (Supplementary Table 2). Among those mRNAs, we selected *TCF1, MYB HHO2*, and *DREB2F*. Besides, we included the cold-responsive markers, *DREB1A* and *COR15A*, that did not show translational changes in 15-day-old *eif(iso)4e-1* mutant seedlings grown under normal temperature conditions and short-day photoperiod. Their levels and those of *eIF4E1* and *eIF(iso)4E* mRNAs were quantified by RT-qPCR in rosette leaves from NAC or AC plants (Figure 4). AC plants showed significantly increased levels of both *eIF4E1* and *eIF(iso)4E* and of *COR15A* and the potential eIF(iso)4E translational target *TCF1*, which displayed about thirty-fold higher levels after acclimation. Available global expression values for the analyzed genes corresponded to short, 24 h cold exposure (Supplementary Table 2). Therefore, the expected rise in *MYB HHO2, DREB2F*, and *DREB1A* expression might have occurred at earlier times and then returned to basal levels. However, our analysis confirmed that *eIF4E1* and *eIF(iso)4E* gene expression was also stimulated at longer exposures to low temperatures and different plant developmental stages.

### Polysome Profiles, and *eIF4E1* and *eIF(iso)4E* mRNA Recruitment to P Under NAC and AC Conditions

**Figure 5:**
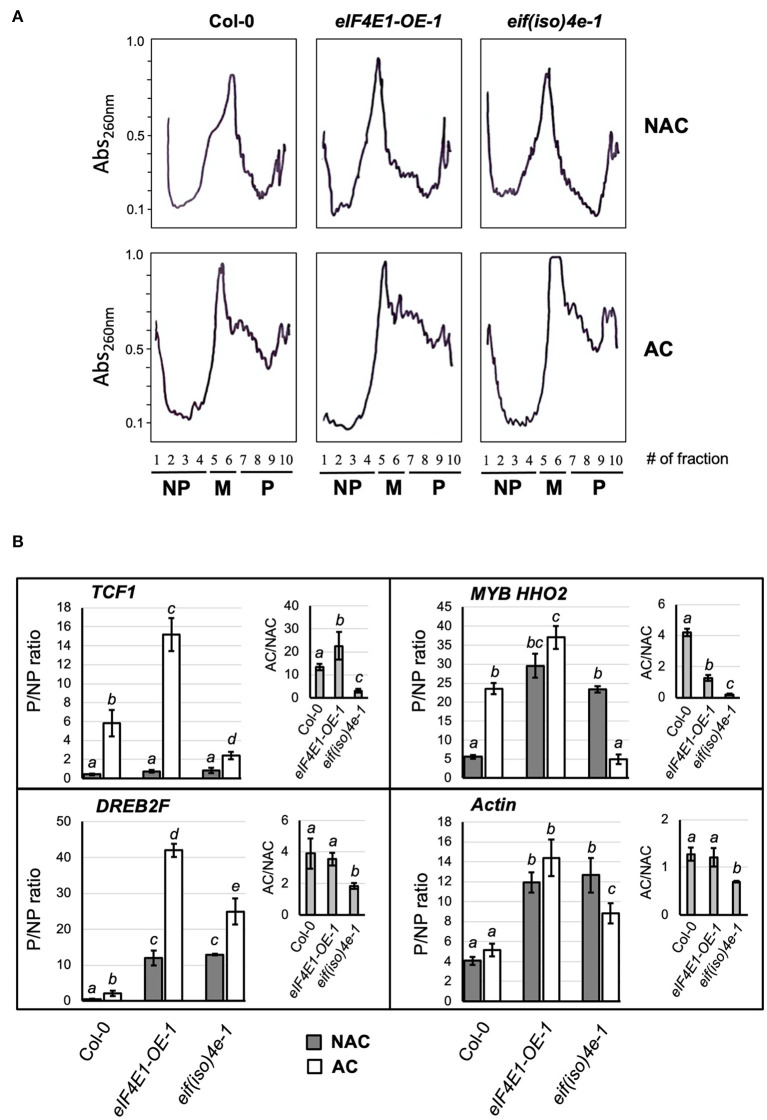
Translation readjustments during plant acclimation to cold temperatures in Col-0 and mutant plants. **(A)** Sucrose gradient sedimentation profiles. Ribosomal fractions from *Arabidopsis thaliana* acclimated (AC) and non-acclimated (NAC) 8-week-old adult plants grown under short photoperiod were fractionated on 20−60% sucrose gradients and collected in 10 fractions under fixed parameters with continuous monitoring of Absorbance at 260 nm (solid line). According to the profiles, fractions 1–4 were considered as non-polysomal (NP), 5–6 as monosomal (M), and 7–10 as polysomal (P). **(B)** The abundance of mRNAs corresponding to stress-responsive genes was analyzed in NP and P pooled fractions from the sucrose gradient sedimentation profiles shown in **(A)**. Gray bars refer to non-acclimated plants; white bars refer to plants acclimated for 1 week at 4°C. Data correspond to RT-qPCR, where P was referenced to NP and normalized by elongation factor *eEF-1A* for each line and condition (2^−ΔΔCt^). Then P/NP ratio was calculated for two independent profiles with three technical replicates and shown as mean values with standard deviation. The AC/NAC ratios for the three lines are represented in the inset graph. **Col-0**, wild-type plants; ***eIF4E1-OE-1***, plants overexpressing eIF4E1; ***eif(iso)4e-1***, plants knockout for eIF(iso)4E.

The enhanced eIF4E1 and eIF(iso)4E expression found at 4°C indicates that both factors might participate in translation regulation to ensure plant adaptation to cold temperatures. Stressful environments have been related to general translation inhibition and the promotion of selective translation for stress-related proteins. In our model, cold acclimation treatment (7 days of exposure) englobes more than the initial stress perception (first hours of exposure). It rather reflects the ability of the plant to continue with its life cycle through a sustained environmental change and may, therefore, include adaptation mechanisms. The polysomal profiles obtained from NAC and AC Arabidopsis rosette leaves corresponding to Col-0 and mutant lines presented distinctive shapes upon acclimation (Figure 5A). The profiles were overlapped to classify fractions as NP (1–4), monosomes (5–6), and P (7–10).

Fractions corresponding to P appeared enriched in AC plants, and the pattern observed for NAC plants was essentially similar for all lines. Therefore, varying eIF4E1 or eIF(iso)4E levels did not affect global translation under control growth conditions, which is consistent with our previous report (Martinez-Silva et al., 2012).

The polysomal distribution of *eIF4E1* and *eIF(iso)4E* mRNAs indicated active translation of both factors under NAC and AC conditions in Col-0 and mutant plants (Supplementary Figure 4). However, a shift toward heavier polysomal fractions (9–10) was observed for *eIF4E1* mRNA in AC (31%) vs. NAC (18%) Col-0 plants. This is consistent with the previously observed increase at the protein level for this factor upon exposure at 4°C (Figure 1). Interestingly, *eIF(iso)4E* displayed an evident enrichment in the same fractions for both NAC and AC *eIF4E1-OE-1* plants (51 and 49%, respectively) as compared to Col-0 (26 and 39%, respectively), suggesting that eIF4E1 overexpression might promote *eIF(iso)4E* translation, at least for the line analyzed in this study (Supplementary Figure 4).

### The Distribution of Several Cold Stress-Responsive mRNAs Across P Changes Upon Acclimation and in the *eIF4E1-OE-1* Line

For an initial exploration on the polysomal distribution of cold-responsive transcripts that potentially could be affected by the eIF4E1 and/or eIF(iso)4E levels, we performed end-point RT-PCR for each collected fraction, except fraction #1 (Supplementary Figure 5). While the *COR15A* transcript was easily detected in either NAC or AC profiles, *MYB HHO2* and *DREB2F* were much less abundant, and *TCF1* was nearly undetectable for the NAC condition. Therefore, the *COR15A* transcript was translated in the absence of cold exposure at least for the developmental stage used in this study (8-week-old rosette leaves). Furthermore, *COR15A* did not show translational enhancement in AC plants (Supplementary Figure 6) despite the observed transcript increase (Figure 4). According to this observation, we did not further test *COR15A* by RT-qPCR. TCF1 was preferentially distributed into P upon acclimation, particularly in *eIF4E1-OE-1* plants (Supplementary Figures 5, 6). Upon acclimation, an apparent enrichment in P was detected for *DREB2F* in *eIF4E1-OE-1* (Supplementary Figures 5, 7).

To determine more accurately whether low-abundant cold stress-responsive transcripts were shifted to translationally active or inactive fractions in the absence of eIF(iso)4E or overexpression of eIF4E1, we pooled fractions 2–4 as NP and 7–10 as P and performed quantitative RT-PCR in two additional independent biological replicates (Figure 5B). Transcript amount in P and NP for each line and condition was first normalized to *eEF1A*, which showed similar distribution for each pool and was not affected importantly by acclimation or the level of eIF4E1 and eIF(iso)4E expression. To determine P/NP, we considered the NP value for each transcript, condition, and line, as a reference sample. The mean values and statistics of P/NP ratios from two biological replicates with three technical replicates each are shown in the middle panels of Figure 5B. To determine the changes in polysomal recruitment due to acclimation, the P/NP in AC was divided by P/NP in NAC for each value corresponding to two biological replicates with three technical replicates, and mean values with statistics are shown in the upper right graph from Figure 5B (AC/NAC). To determine the potential effect of eIF(iso)4E absence or eIF4E overexpression on the translation of mRNAs corresponding to housekeeping genes, we also evaluated the distribution of *Actin* as a transcript preferentially loaded in P.

*TCF1* was translationally enhanced in *eIF4E1-OE-1* and reduced in *eif(iso)4e-1* plants during AC when compared to wild-type Col-0. For *DREB2F*, the ratio of polysome association in AC/NAC was maintained in the *eIF4E1-OE-1* and reduced in the *eIF(iso)4e-1* mutant compared to Col-0. However, there was still an increase in *DREB2F* mRNA level in the P of *eif(iso)4e-1* mutant in AC plants. On the other hand, *MYB HHO2*, which was mostly present in P, showed a decreased ratio of polysome association in AC/NAC in both *eIF4E1-OE-1* and *eif(iso)4e-1* mutants as compared to Col-0 (Figure 5B; Supplementary Figures 5, 7). According to these results, we concluded that *TCF1* translation was favored by higher levels of eIF4E1 in *eIF4E1-OE-1*, but not in *eif(iso)4e-1*, plants. This, along with the differences in polysome recruitment ratios between AC and NAC of *DREB2F* and *MYB HHO2*, suggests that the concerted action of both isoforms is required to elicit appropriate responses related to translational adjustments of particular cold-responsive transcripts when a decrease in temperature occurs.

## Discussion

Expression of stress-responsive genes is crucial when the plant faces unfavorable conditions and is regulated at several points (Chinnusamy et al., 2007; Ding et al., 2020). A series of *cis*-acting regulatory elements have been identified within the promoters of stress-responsive genes. These include ABA-responsive element (ABRE), MYC and MYB recognition sites, ethylene-responsive element (ERE), ICE-box (recognized by an inducer of CBF expression factors), anaerobic-responsive element (ARE), dehydration-responsive element (DRE), low-temperature-responsive element (LTRE), and heat shock element (HSE) among others (Yamaguchi-Shinozaki and Shinozaki, 2005). An additional layer of regulation is exerted by translation adjustments under stress as an immediate and effective response to maintain cellular homeostasis. Due to the high cost of protein synthesis, selective translation becomes particularly important under stress (Bailey-Serres et al., 2009; Echevarria-Zomeno et al., 2013). Initial stress perception has been related to lowering protein synthesis rates and growth decrease. However, in the case of low temperatures, continuous exposure triggered mechanisms favoring translation to allow Arabidopsis plants not only to contend with stress but also to continue their development under such conditions (Beine-Golovchuk et al., 2018; Garcia-Molina et al., 2020; Xi et al., 2020). In this sense, we observed a polysomal increase in the sucrose gradient profiles from 1-week-AC plants at 4°C, confirming that translation was activated upon long-term cold exposure.

Interestingly, both, *eIF4E1* and *eIF(iso)4E*, promoters display stress response-related *cis*-acting elements (Supplementary Table 3). Particularly, the *eIF4E1* promoter shows stress-related hormone-responsive elements also found in cold- and dehydration stress-responsive genes. This is consistent with the upregulated expression of both factors under cold exposure in Col-0 plants. In addition, enhanced *eIF4E1* and *eIF(iso)4E* transcript recruitment to translationally active fractions and increased protein levels were observed. Such behavior suggests that these cap-binding factors participate in stress response to possibly overcome further oncoming harsh conditions and supports a modulated translation according to plant requirements. Interestingly, from the cold response-related transcripts tested in this study, *COR15A* and *TCF1* remained highly expressed in AC plants, while *DREB1A, DREB2F*, and *MYB HHO2* showed transcript levels similar to the control plants. This confirmed that some transcriptional responses operate transiently or by rounds during cold acclimation.

Arabidopsis eIF4E1 and eIF(iso)4E could function by promoting selective transcript recruitment to translation complexes or transcript stability and storage in response to low temperatures, in accordance with the well-described gene expression modules enabling freezing tolerance (Thomashow, 1999; Knight and Knight, 2012). We tested the effect of eIF(iso)4E absence/presence and eIF4E1 overexpression on polysomal recruitment of cold-responsive transcripts *COR15A* and *TCF1* that are significantly enriched upon low temperature exposure. *TCF1* was reduced in polysomal fractions in the *eif(iso)4e-1* KO mutant during AC but enhanced in the eIF4E1 overexpressing line, while *COR15A* seems to remain uniformly distributed along the profiles of all lines, at least in this latter case according to the semi-quantitative measurements performed in two different profiles. This suggests that transcripts activated by cold show distinct dependency on eIF4E factors and present specific translation adjustments upon temperature drop. *COR15A* forms part of genes activated by C-repeat-binding transcription factors (CBF) under low temperatures, whereas *TCF1* belongs to a CBF-independent pathway (Ji et al., 2015). *TCF1* was previously found as a translationally affected transcript in the *eif(iso)4e-1* mutant (Martinez-Silva et al., 2012).

Another transcript showing altered translation in the absence of eIF(iso)4E was *MYB-HHO2* and, to a lesser extent, *DREB2F*. In spite of not being particularly related to cold exposure, these have been described as abiotic stress-related genes (Mizoi et al., 2012; Nagarajan et al., 2016). *DREB2F* shift to polysomal fractions in AC/NAC was reduced in the *eif(iso)4e-1* KO mutant, while a reduction in this value was observed in *eIF4E1-OE-1* and *eif(iso)4e-1* lines in the case of *MYB HHO2*. This suggests that precise levels of eIF(iso)4E and/or eIF4E1 are required for proper translation of these transcripts during acclimation.

The polysomal distribution changes observed for the tested genes contrasted to *COR15A* and the control transcript *Actin*, supporting selective translation due to long exposure to low temperatures and differential dependency on eIF(iso)4E and eIF4E1 cap-binding factors. Since the recruitment of *eIF(iso)4E* to heavy polysomal fractions was more prominent compared to *eIF4E1* and appeared further stimulated in *eIF4E1-OE-1* plants, even in the absence of cold, it is possible that this isoform is relevant to translate particular mRNA pools in response to stress. Several transcripts previously found as translationally affected in the *eif(iso)4e-1* KO mutant appeared stimulated at low temperatures (Supplementary Table 2). Some of them have been related to cell wall flexibility, membrane compartment stabilization, osmotic equilibrium maintenance, and antioxidant mechanisms (Mizoi et al., 2012; Ji et al., 2015; Dutta et al., 2020). Particularly, the *TCF1* translational behavior in mutant lines is consistent with the observed phenotypes in response to freezing temperatures. TCF1 regulates freezing tolerance and cold acclimation by promoting specific transcriptional programs involved in cell wall remodeling and growth regulation. In addition, the translational status of transcription factors modulating stress-responsive gene expression, such as *MYB HHO2* and others, not studied in this work, could account for the observed behavior. Massive RNA sequencing of translationally active and inactive fractions or ribosome profiling under cold conditions in *eif(iso)4e-KO* and *eif4e1-KO* lines compared to wild-type Col-0 would help to enrich the picture of translational control exerted by these factors in cold responses. In addition, different eIF4E1 overexpressing lines must be tested to confirm the effect of increased eIF4E1 levels on candidate transcript translation.

Studies on eIF(iso)4E mutant in Arabidopsis have suggested partially redundant functions with eIF4E1 in plant development (Duprat et al., 2002) as opposed to its partner eIF(iso)4G, where the corresponding mutant lines displayed impaired growth and viability, enhanced susceptibility to dehydration and salinity (Lellis et al., 2010). In the absence of eIF(iso)4E, eIF4E1 showed increased protein levels, mostly due to higher abundance of the mRNA in polysomal fractions and no change at the transcript level (Duprat et al., 2002). However, closer inspection on translation activity showed disturbances for specific transcripts in the absence of eIF(iso)4E, related to root growth and phosphate transport (Martinez-Silva et al., 2012).

According to the results shown here, both eIF4E1 and eIF(iso)4E are required for basal freezing tolerance in Arabidopsis adult plants. The higher eIF4E1 levels observed in eIF(iso)4E null background are apparently unable to substitute the function of the other isoform, but in the presence of eIF(iso)4E would favor increased freezing tolerance. Interestingly, for AC plants there were no differences in freezing tolerance between Col-0 wild-type and mutant plants. A possible explanation is that after extended exposure to low temperatures, several regulatory mechanisms become operational to enable appropriate plant freezing response. On the other hand, basal levels of cold-related transcription factor translation could depend on selective action/levels of eIF4E1 and eIF(iso)4E in plant tissues for early responses to cold exposure as predicted by their expression stimulation. In this sense, it would be worth to explore freezing tolerance and translation changes at earlier times of low-temperature exposure. Therefore, different acclimation and freezing exposure times might be worth to assay in the future to access variable responses in *eIF4E1-OE* plants.

## Data Availability Statement

Publicly available datasets were analyzed in this study. This data can be found here: http://www.ncbi.nlm.nih.gov/geo/query/acc.cgi?acc=GSE29386.

## Author Contributions

KS-D, EH-L, MG-R, and TD conceived the idea and designed the experiments. KS-D, MA-L, EH-L, BN-R, MP-T, and JJ-P performed the experiments. KS-D, MA-L, EH-L, BN-R, MP-T, and TD analyzed the data. KS-D, MA-L, and TD wrote the paper. All authors revised the final version.

## Funding

This work was funded by Consejo Nacional de Ciencia y Tecnología: 238439, Universidad Nacional Autónoma de México: PAPIIT IN214118, 218921, IN222815, IN220618, and Facultad de Química PAIP 5000-9118. Both institutions had no role in the study design, collection, analysis, or interpretation of the data, writing the manuscript, or the decision to submit the paper for publication.

## Conflict of Interest

The authors declare that the research was conducted in the absence of any commercial or financial relationships that could be construed as a potential conflict of interest.

## Publisher's Note

All claims expressed in this article are solely those of the authors and do not necessarily represent those of their affiliated organizations, or those of the publisher, the editors and the reviewers. Any product that may be evaluated in this article, or claim that may be made by its manufacturer, is not guaranteed or endorsed by the publisher.
