# Supplementary material for: Arabidopsis thaliana eIF4E1 and eIF(iso)4E Participate in Cold Response and Promote Translation of Some Stress-Related mRNAs
Source: Front Plant Sci. 2021 Sep 30;12:698585. doi: 10.3389/fpls.2021.698585 (PMC8514651; doi:10.3389/fpls.2021.698585)
Supplement: Supplementary file 1 [file Data_Sheet_1.PDF]

Supplementary Material

1 Supplementary Figures and Tables

1.1 Supplementary Figures

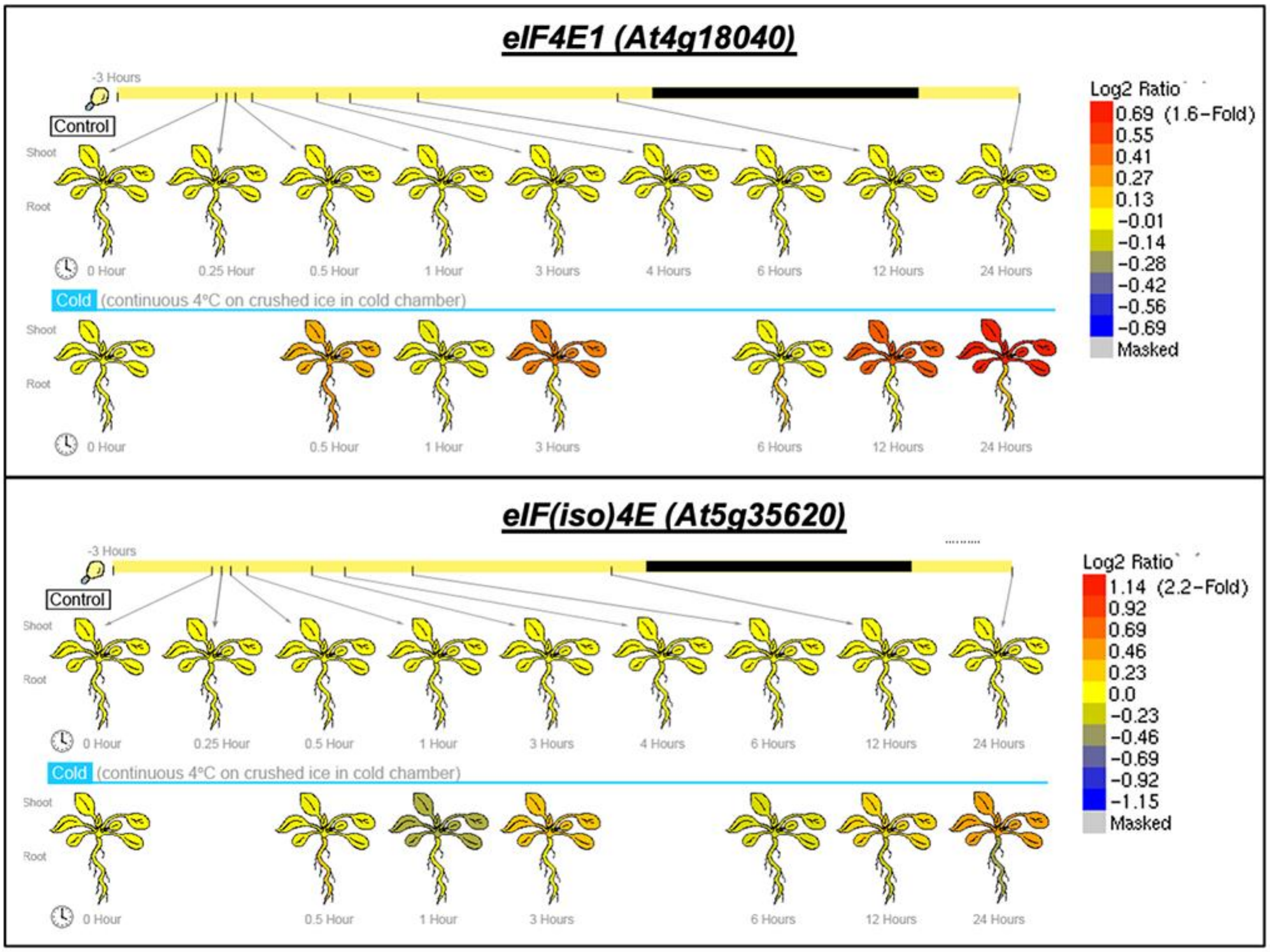

Supplementary Figure S1. Expression levels of *Arabidopsis thaliana eIF4E1* and *eIF(iso)4E* genes in response to cold, according to global RNA analyses (www.bar.utoronto.ca). Data were obtained for 15 days-old seedlings exposed (lower row of each panel) or not (upper row of each panel) to 4 ºC under long photoperiod as indicated by the bar. Different time points of exposure are indicated.

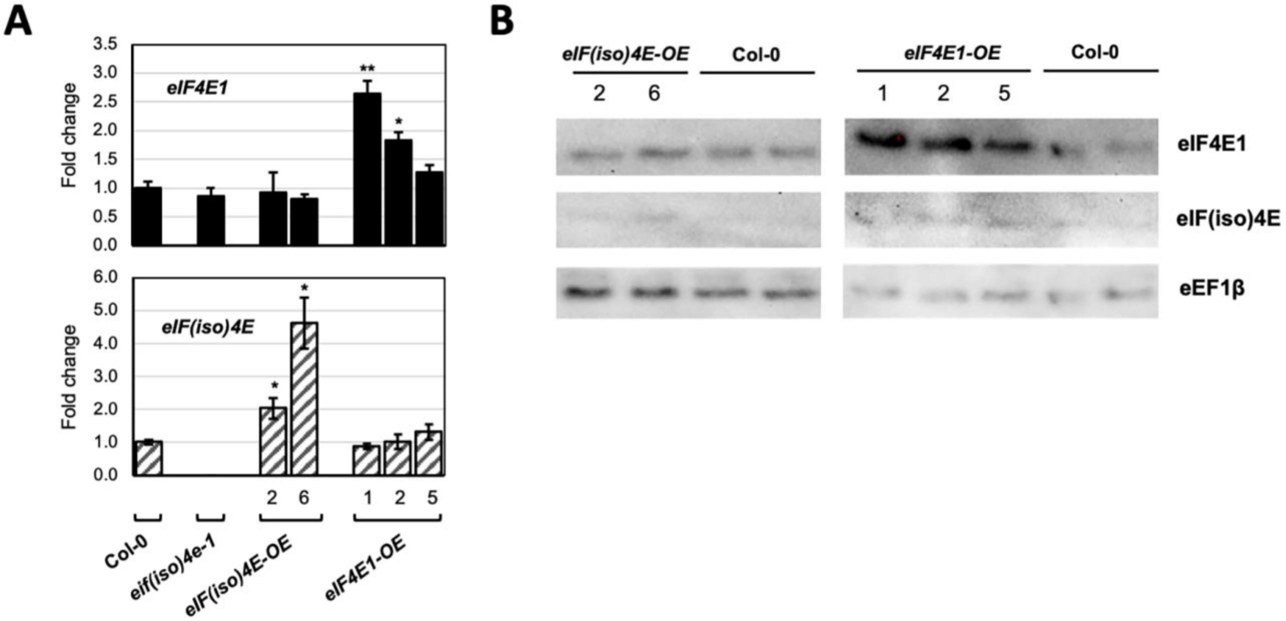

Supplementary Figure S2. Expression levels of eIF4E1 and eIF(iso)4E in transgenic *Arabidopsis thaliana* lines. A) Transcript levels were evaluated by RT-qPCR for each, *eIF4E1* and *eIF(iso)4E* transcripts, in adult 5 weeks-old rosette leaves from the F4 generation of different lines transformed with *35S:eIF4E1* [*eIF4E1-OE*] or *35S:eIF(iso)4E* [*eIF(iso)4E-OE*] as compared to wild type (Col-0). The 18S rRNA was used as loading control. Student's t-test was used to evaluate statistically significant differences: (*) $p < 0.05$ (**) $p < 0.01$. **B)** Protein levels were evaluated by western blot on total protein extracts from lines showing higher RNA accumulation than wild type (Col-0). The eIF1β was used as loading control. Line 1 for ***eIF4E1-OE*** and line 6 for ***eIF(iso)4E-OE*** were used in all subsequent experiments.

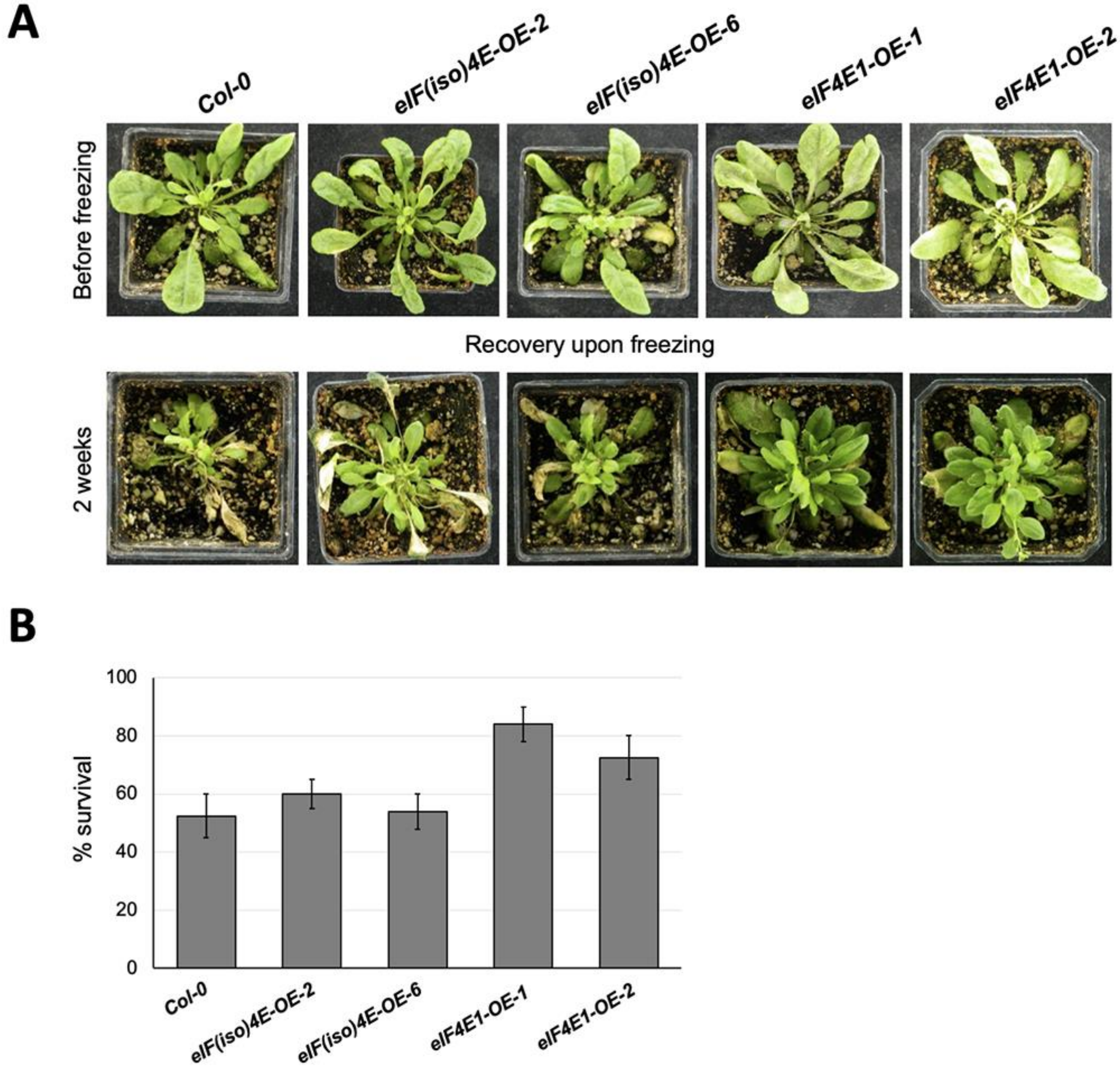

Supplementary Figure S3. Pilot experiment for phenotyping eIF4E1 and eIF(iso)4E overexpressing lines in response to freezing temperatures. A. Plant phenotypes were registered for 7 weeks-old plants grown at 22 ºC under 8 h light/16 h darkness photoperiod (short day) before freezing, and at 2 weeks after exposure to freezing temperatures (-20 ºC) for 1 h. **B.** Percentage of plant survival upon freezing without acclimation (results from 2 pilot experiments with 5 plants per line). Col-0, wild type; *eIF(iso)4E-OE*, lines 2 and 6, plants expressing eIF(iso)4E under the CaMV 35S promoter; *eIF4E1-OE*, lines 1 and 2, plants expressing eIF4E1 under the CaMV 35S promoter.

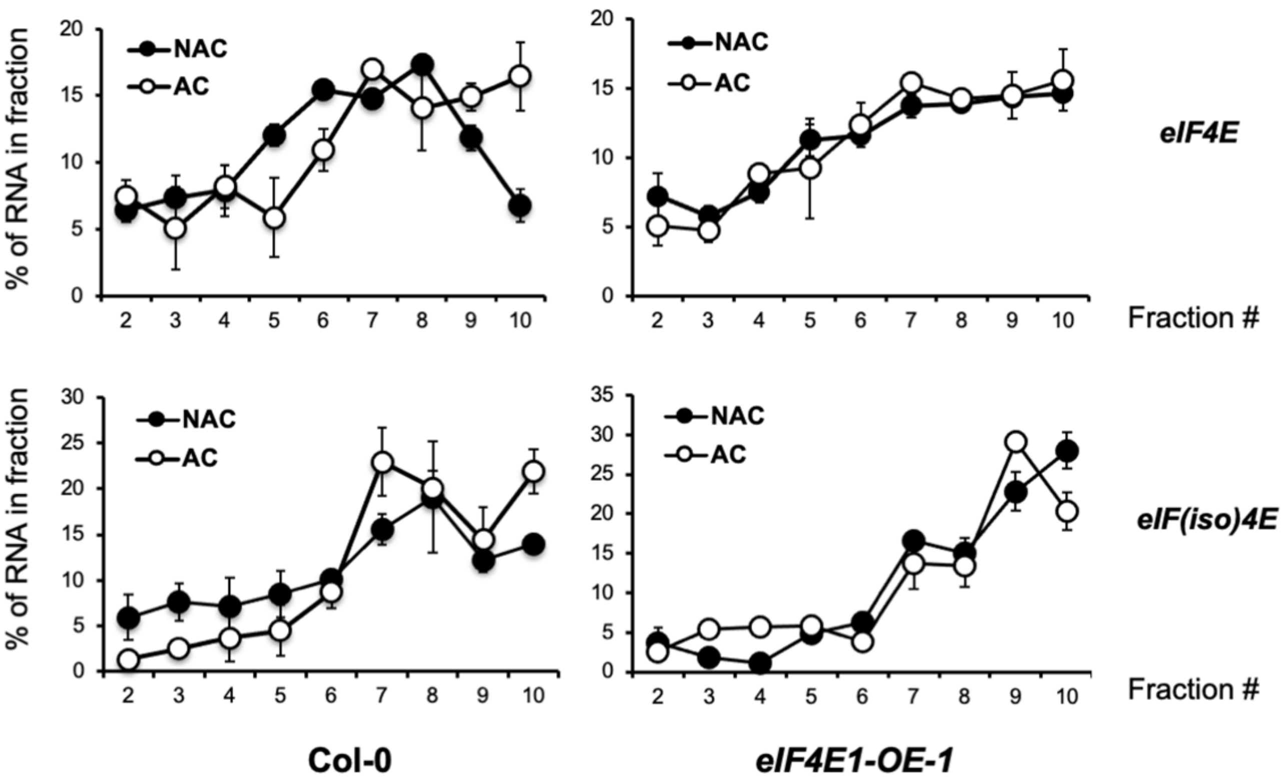

Supplementary Figure S4. Distribution of *eIF4E1* and *eIF(iso)4E* mRNA across polyosomal profiles under acclimation. Transcript RNA levels were represented as percentage corresponding to each fraction (numbers on the abscise) from the sum of all fractions. Data were obtained as average of semi-quantitative RT-PCR analysis in two independent biological replicates. Bars represent standard deviation. **Col-0**, wild type plants; ***eIF4E1-OE-1***, plants overexpressing eIF4E1; **NAC**, non-acclimated; **AC**, acclimated.

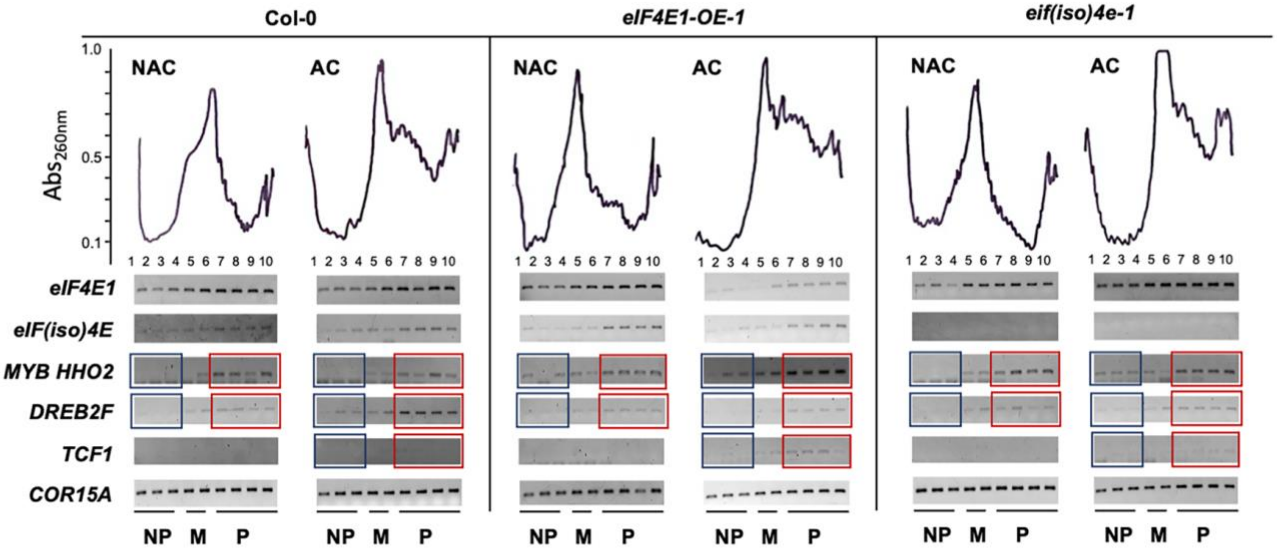

Supplementary Figure S5. Transcript distribution across polysome profiles from wild type and mutant plants. RNA isolated from each fraction, corresponding to the upper polysome profile, was subjected to RT-PCR as described in methods. According to the profile, fractions were identified as non-polysomes (**NP**), monosomes (**M**) and polysomes (**P**). Fraction 1 was omitted for all analyses. **Col-0**, wild type plants; ***eIF4E1-OE-1***, plants overexpressing eIF4E; ***eif(iso)4e-1***, plants knockout for eIF(iso)4E; **NAC**, non-acclimated; **AC**, acclimated.

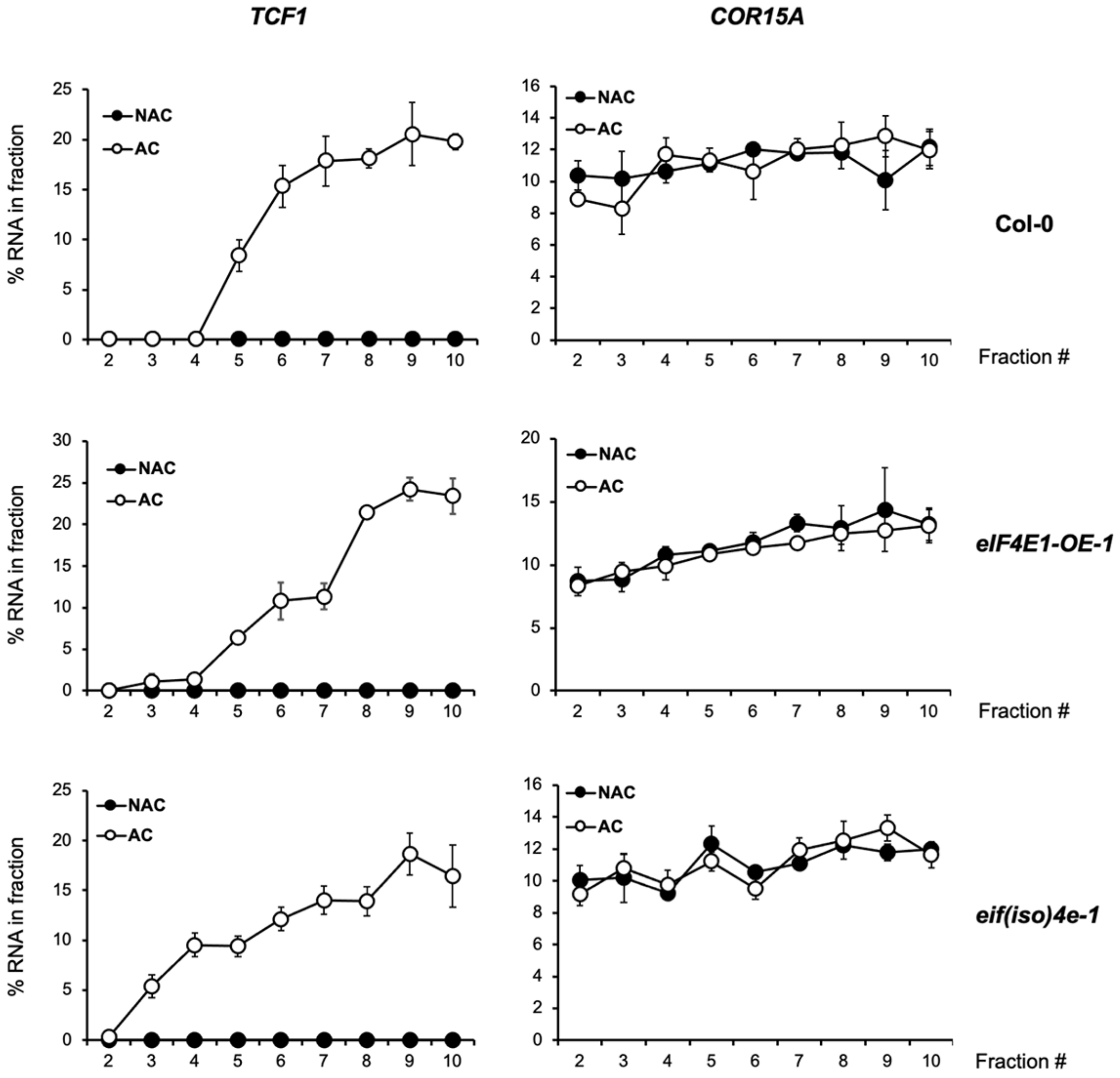

Supplementary Figure S6. Distribution of *TCF1* and *COR15A* mRNA across polyosomal profiles under acclimation. Transcript RNA levels were represented as percentage corresponding to each fraction (numbers on the abscise) from the sum of all fractions. Data were obtained as average of semi-quantitative RT-PCR analysis in two independent biological replicates. Bars represent standard deviation. **Col-0**, wild type plants; ***eIF4E1-OE-1***, plants overexpressing eIF4E1; ***eif(iso)4e-1***, plants knockout for eiF(iso)4E; **NAC**, non-acclimated; **AC**, acclimated.

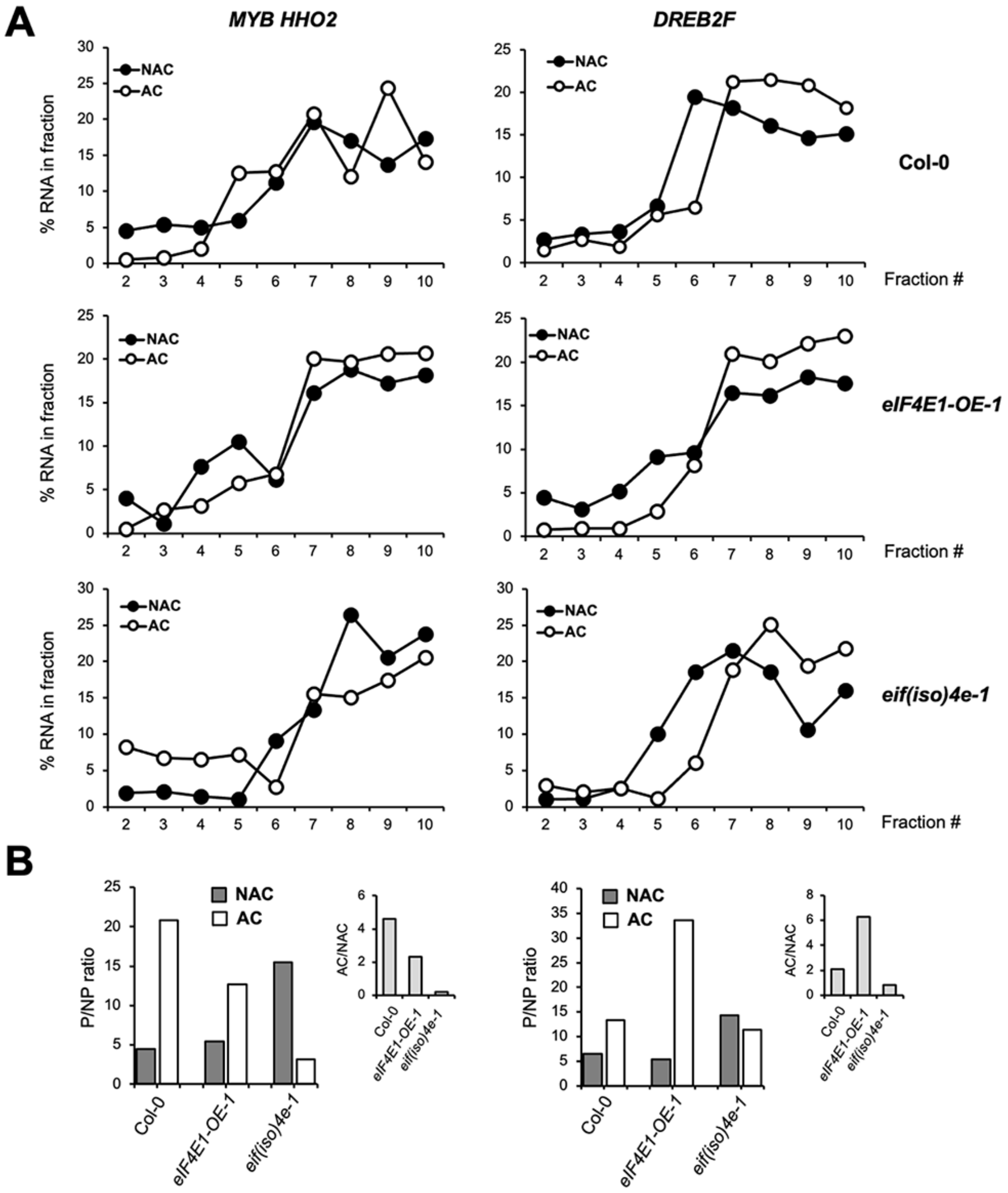

Supplementary Figure S7. Distribution of *DREB2F* and *MYB HHO2* mRNA across polyosomal profiles under acclimation. A. Transcript RNA levels were represented as percentage corresponding to each fraction (numbers on the abscise) from the sum of all fractions. Data were obtained from densitometry of the semi-quantitative RT-PCR analysis shown in Figure S5. **B.** The P/NP ratio was calculated for fractions 7-10 (P) over 2-4 (NP) and plotted for each line and condition. The AC/NAC ratios for the three lines are represented in the inset graph. **Col-0**, wild type plants; ***eIF4E1-OE-1***, plants overexpressing eIF4E1; ***eif(iso)4e-1***, plants knockout for eiF(iso)4E; **NAC**, non-acclimated; **AC**, acclimated.

1.2 Supplementary Tables

Supplementary Table S1. Oligonuclotide sequences used in this study

Gene name	Accession	Oligonucleotide sequence (5’ → 3’)
eIF4E1	*At4g18040*	FW: GGTAGAAGACACTCCCAAATC
		RV: CATCACCTTCCTCATGGTATCG
eIF(iso)4E	*At5g35620*	FW: GAGAAACAACCACACAAGCTC
		RV: CCAAAAATCTTCGACGGTGTC
MYB HHO2	*At1g68670*	FW: ACCGTCCGATGTAGCCAAC
		RV: GCAACTTCTCTCCAACGAACG
DREB2F	*At3g57600*	FW: ACCGTCCGATGTAGCCAAC
		RV: GCAACTTCTCTCCAACGAACG
TCF1	*At3g55580*	FW: ATGACGACCACCAGCAGCAAC
		RV: TCTGACAACATTGACCTCTCGG
DREB1A	*At4g25480*	FW: TGAGATGCCGAGTTTGTTGG
		RV: ATTCCACTGTACGGACGGAAG
COR15A	*At2g42540*	FW: AGGAGAAAGGAAAAGAAGCC
		RV: AAGACCCTACTTTGTGGCATCC
18S rRNA		FW: TCCTATTGTTGGCCTTCGG
		RV: TCCTTGGCAAATGCTTTCGC

Supplementary Table S2. Translationally affected genes in *eif(iso)4e-1* knockout mutant, that have been related to cold stress (Martinez-Silva et al., 2012). Two additional cold-responsive genes, unchanged in *eif(iso)4e-1* were also selected for analysis.

**Translationally down-regulated in *eif(iso)4e-1***		
**Gene ID**	**Function**	**Expression response at 4ºC[a]**
At3g55580	Tolerant to chilling and freezing 1 (TCF) regulator of chromosome condensation (RCC1) family protein	94-fold increase at 12h in leaves
At1g05550	Protein of unknown function (DUF295)	70-fold increase at 24h in leaves
At4g02950	Ubiquitin family protein	4.1-fold increase at 0.5h in roots
At4g09280	Transposable element gene; similar to Ulp1 protease family protein	11-fold decrease at 3h in roots
At2g24762	Glutamine dumper 4 (GDU4), involved in amino acid transport	17-fold decrease at 12h in roots
At1g19020	Small Defense-Associated protein 1 (SDA1), pathogen defense, oxidative stress tolerance	10-fold increase at 24h in roots
**Translationally up-regulated in *eif(iso)4e-1***		
**Gene ID**	**Function**	**Expression response at 4ºC**
At1g68670	Hypersensitivity to low phosphate-elicited Primary root shortening 1 Homolog 2 (HHO2), MYB-related transcription factor	3.3-fold increase at 1h in leaves
At3g57600	AP2-ERF transcription factor drought response element binding (DREB2F)	2.3-fold increase at 1h in roots
At1g66310	F-box/RNI-like/FBD-like domains-containing protein	3-fold decrease a 24h in leaves

At5g15850	Transcription factor Constans-Like 1 (COL1)	13-fold increase at 6h in leaves
**Translationally unchanged in *eif(iso)4e-1***		
At4g25480	AP2-ERF transcription factor drought response element binding (DREB1A)	326-fold increase at 12h in leaves
At2g42540	Cold-regulated 15a (COR15A), chloroplast targeted	421-fold increase at 24h in leaves

[a] Data were taken from Arabidopsis eFP Browser (www.bar.utoronto.ca; (Winter et al., 2007)).

Supplementary Table S3. Stress response *cis* elements in *Arabidopsis thaliana eIF4E1* and *eIF(iso)4E* promoters. The sequences comprising 1500 nt upstream of the transcription initiation sites for *eIF4E1* (*At4g18040*) and *eIF(iso)4E* (*At4g35620*) were obtained from PlantPAN 2.0 (http://plantpan2.itps.ncku.edu.tw) and analyzed with PlantCARE (http://bioinformatics.psb.ugent.be/webtools/plantcare/html).

***cis* element**	**Gene promoter**	**Position**	**Sequence**	**Function**
**ABRE**	*eIF4E1*	-685	GCCACGTACA	Absicic acid response
**ARE**	*eIF4E1*	-646; -394	TGGTTT	Anaerobic response
**ARE**	*eIF(iso)4E*	-1470; -336	TGGTTT	Anaerobic response
**CGTCA motif**	*eIF4E1*	-292	CGTCA	Methyl jasmonate response
**HSE**	*eIF4E1*	-606	AAAAAATTTC	Heat shock response
**MYB**	*eIF4E1*	-519; -776	T/CAACTG	MYB factor- binding site, drought stress related
**ERE**	*eIF(iso)4E*	-538	ATTTCAAA	Ethylene response
**TCA**	*eIF4E1*	-185	GAGAAGAATA	Salicylic acid response

Martinez-Silva, A.V., Aguirre-Martinez, C., Flores-Tinoco, C.E., Alejandri-Ramirez, N.D., and Dinkova, T.D. (2012). Translation initiation factor AteIF(iso)4E is involved in selective mRNA translation in Arabidopsis thaliana seedlings. *PLoS One* 7**,** e31606.

Winter, D., Vinegar, B., Nahal, H., Ammar, R., Wilson, G.V., and Provart, N.J. (2007). An "Electronic Fluorescent Pictograph" browser for exploring and analyzing large-scale biological data sets. *PLoS One* 2**,** e718.
